# Bioprinted liver provides early insight into the role of Kupffer cells in TGF-β1 and methotrexate-induced fibrogenesis

**DOI:** 10.1371/journal.pone.0208958

**Published:** 2019-01-02

**Authors:** Leah M. Norona, Deborah G. Nguyen, David A. Gerber, Sharon C. Presnell, Merrie Mosedale, Paul B. Watkins

**Affiliations:** 1 Curriculum in Toxicology, University of North Carolina at Chapel Hill, Chapel Hill, North Carolina, United States of America; 2 Division of Pharmacotherapy and Experimental Therapeutics, UNC Eshelman School of Pharmacy, Chapel Hill, North Carolina, United States of America; 3 The Institute for Drug Safety Sciences, University of North Carolina at Chapel Hill, Research Triangle Park, North Carolina, United States of America; 4 Research and Development, Organovo, Inc., San Diego, California, United States of America; 5 Department of Surgery, University of North Carolina at Chapel Hill, Chapel Hill, North Carolina, United States of America; 6 Department of Pathology & Laboratory Medicine, University of North Carolina at Chapel Hill, Chapel Hill, North Carolina, United States of America; National Institutes of Health, UNITED STATES

## Abstract

Hepatic fibrosis develops from a series of complex interactions among resident and recruited cells making it a challenge to replicate using standard *in vitro* approaches. While studies have demonstrated the importance of macrophages in fibrogenesis, the role of Kupffer cells (KCs) in modulating the initial response remains elusive. Previous work demonstrated utility of 3D bioprinted liver to recapitulate basic fibrogenic features following treatment with fibrosis-associated agents. In the present study, culture conditions were modified to recapitulate a gradual accumulation of collagen within the tissues over an extended exposure timeframe. Under these conditions, KCs were added to the model to examine their impact on the injury/fibrogenic response following cytokine and drug stimuli. A 28-day exposure to 10 ng/mL TGF-β1 and 0.209 μM methotrexate (MTX) resulted in sustained LDH release which was attenuated when KCs were incorporated in the model. Assessment of miR-122 confirmed early hepatocyte injury in response to TGF-β1 that appeared delayed in the presence of KCs, whereas MTX-induced increases in miR-122 were observed when KCs were incorporated in the model. Although the collagen responses were mild under the conditions tested to mimic early fibrotic injury, a global reduction in cytokines was observed in the KC-modified tissue model following treatment. Furthermore, gene expression profiling suggests KCs have a significant impact on baseline tissue function over time and an important modulatory role dependent on the context of injury. Although the number of differentially expressed genes across treatments was comparable, pathway enrichment suggests distinct, KC- and time-dependent changes in the transcriptome for each agent. As such, the incorporation of KCs and impact on baseline tissue homeostasis may be important in recapitulating temporal dynamics of the fibrogenic response to different agents.

## Introduction

Liver fibrosis poses an important human health concern from both a clinical and regulatory standpoint. Because fibrosis develops over time from a sequence of complex and cumulative interactions between hepatocytes and non-parenchymal cells (NPCs), it has proven challenging to replicate using standard *in vitro* and preclinical *in vivo* models. Crosstalk among hepatocytes and resident NPCs normally maintains a balance in reparative processes following injury, such as inflammatory cytokine release, response to oxidative stress, and synthesis/change in extracellular matrix (ECM) composition. Fibrosis is marked by an imbalance in these processes and can progress to compromise liver function via changes in the tissue microenvironment, production of various growth factors, inflammatory cytokines, and disruption of normal liver architecture as a result of a change in the distribution and proportion of fibrillar collagens [1]. The inflammatory response plays an important role in driving these processes, as resident and recruited extrahepatic inflammatory cells are thought to create a more conducive environment via the production of inflammatory and fibrogenic mediators that further amplify the response [2, 3].

In uninjured liver, Kupffer cells (KCs) constitute the main population of inflammatory cells and are important for a number of homeostatic functions [4, 5]. During chronic injury, extrahepatic inflammatory cells are recruited to the site of damage and dramatically shift the population of immune cells in the liver [6]. While studies utilizing transgenic approaches or selective blockade have demonstrated the general importance of macrophages in not only the initiation of fibrotic injury but also its resolution, the specific role of KCs during early injury as one of the initial responders remains elusive [7, 8]. This is largely due to the inability to specifically target subset populations (*i*.*e*., resident versus recruited) *in vivo* and the heterogeneity of macrophages during liver injury [6].

Previous work has demonstrated the utility of a 3D bioprinted liver tissue model (ExVive 3D Human Liver, Organovo) comprised of primary human hepatocytes, hepatic stellate cells (HSCs), and endothelial cells (ECs) to recapitulate basic fibrogenic features following treatment with prototype fibrogenic agents [9]. While the standard model lacks KCs, the bioprinting process confers a unique advantage by enabling the controlled incorporation of these cells in an automated and precise fashion. Hence, the present study sought to understand the role of resident KCs in mediating the injury and fibrogenic response using a modified model based on previous studies [9]. Our results suggest KCs impact different phases of the response on a biochemical and gene expression level and that their modulation of the injury/fibrogenic response is context dependent. Thus, we further demonstrate bioprinted human liver tissues are well-suited to model and examine temporal fibrogenic events *in vitro* and their utility in beginning to understand the early events underlying fibrotic injury.

## Materials and methods

### Tissue production

Three-dimensional bioprinted liver tissues comprising primary cryopreserved human hepatocytes (Life Technologies), HSCs (ScienCell), and human umbilical vein endothelial cells (ECs) (Becton Dickinson) were manufactured by Organovo with (modified model) and without (standard model) the incorporation of primary human KCs (Samsara Sciences) at an estimated physiologically relevant ratio as described previously [9–14]. Briefly, HSCs and ECs were cultured prior to tissue fabrication and cryopreserved. Hepatocytes and KCs were thawed and prepared for use according to the manufacturer’s instructions. Separate bio-inks comprising parenchymal cells with or without KCs (100% cellular paste, generated via compaction) or NPCs (150e^6^ HSCs + ECs/mL formulated in NovoGel 2.0 Hydrogel) were prepared and loaded into separate heads of the NovoGen Bioprinter platform (Organovo). An automated computer script was then executed to precisely deposit the bio-inks in a two-compartment planar geometry onto the membranes of standard 24-well 0.4 mm transwell membrane inserts (Corning, Tewksbury, Massachusetts) via continuous deposition, with HSCs and ECs comprising the border regions of each compartment and hepatocytes with or without KCs filling each compartment. Cell ratios in the final printed construct roughly approximated physiologic ratios and the tissue thickness was approximately 500 mm. Sixty hours post-fabrication, tissues were transitioned in dexamethasone-free William’s E medium supplemented with Primary Hepatocyte Maintenance Supplements (Life Technologies) and EGM-2 (Lonza) and maintained in a 37°C incubator under humidified atmospheric conditions with 5% CO_2_. Liver constructs were allowed to mature into tissue-like structures for a minimum of six days post-manufacture with the daily replacement of medium prior to the initiation of treatment.

### Compound exposure

LC20 and LC50 concentrations of methotrexate (MTX; Sigma-Aldrich) identified based on 14-day dose-response studies in the standard tissue model (S1 Fig.) were used to conduct the present studies. All dosing solutions were prepared immediately prior to addition to liver tissue constructs. Stock concentrations of MTX prepared in 100% dimethyl sulfoxide (DMSO; Sigma-Aldrich) were diluted in 3D Liver Tissue Medium without dexamethasone (Organovo) to a final concentration of 0.052 μM and 0.209 μM (final DMSO concentration, 0.1%). Lyophilized TGF-β1 was reconstituted in Corning USP/EP Certified Sterile WFI-Quality Water (Fisher Scientific) according to the product data sheet recommendations and added to the medium to prepare dosing solutions, as described previously [9]. TGF-β1 was used at both an estimated physiologically relevant concentration (0.1 ng/mL) and at a concentration traditionally employed in *in vitro* model systems (10 ng/mL) as a positive control [15]. To ensure vehicle consistency across treatment groups, 100% DMSO was spiked into the TGF-β1 dosing solution and standard culture medium (vehicle control) such that the final DMSO concentration was 0.1%. Medium was changed and fresh compound applied daily for 14 or 28 days starting on Day 7 post-manufacture.

### Biochemical assessment of tissue viability and function

#### Lactate dehydrogenase

Medium samples collected on alternate treatment days were analyzed on the day of collection for lactate dehydrogenase (LDH) activity using a commercially available colorimetric assay (Abcam) with modifications as described previously [9].

#### miR-122

miR-122 was assayed using qPCR as described previously [16] in combination with a method described by Miousse, *et al*. to measure miR-122 directly from cleared medium samples with some modifications [17]. Briefly, a standard curve with known concentrations of miR-122 was generated using a synthetic miScript miRNA mimic (Qiagen). Pre-reverse transcription (RT) reactions for each standard and sample were prepared in parallel using miRNA-specific stem loop primers for miR-122 (Applied Biosystems) with subsequent RT reactions performed using the TaqMan miRNA Reverse Transcription Kit (Applied Biosystems). A preamplification step was performed for each RT reaction immediately prior to qPCR using the TaqMan PreAmp Master Mix (Applied Biosystems). qPCR was performed on all undiluted preamplification products using the TaqMan Universal PCR Master Mix II with no *uracil-N-glycosylase* and miRNA-specific TaqMan primer/probe mixes (Applied Biosystems). Triplicate reactions were carried out on a 7900HT Fast Real-Time PCR System (Applied Biosystems). Absolute miR-122 copy number was then interpolated from a standard curve.

#### Albumin and urea

Medium samples collected from treatment days 14 and 28 were analyzed for albumin content using a plate reader-based sandwich ELISA (Bethyl Laboratories) and urea production using a colorimetric assay (BioVision Incorporated). Assays were conducted per the manufacturer’s instructions with minor modifications as described previously [9]. Biochemical data have been deposited in the Dryad Digital Repository and can be downloaded at doi: 10.5061/dryad.5rc1973.

### Histology

Due to the nature of the study, separate tissue manufacturing runs were performed per tissue composition to acquire 14- and 28-day terminal endpoints. The overall LDH response to treatment during the initial half of the exposure period was consistent across 14- and 28-day prints regardless of tissue composition (S2 Fig). A subset of bioprinted liver tissues from each treatment group at each timepoint were formalin-fixed, processed, embedded, and sectioned at a 5.0 μm thickness using a rotary microtome (Jung Biocut 2035; Leica Biosystems) as described previously [9]. Sections were stained with Gomori’s One-Step trichrome (American MasterTech) to visualize collagen content. Slides were imaged using the Aperio AT2 Digital Slide Scanner (Leica Biosystems). For immunohistochemistry, formalin-fixed bioprinted liver tissue sections were deparaffinized and subject to heat-mediated antigen retrieval in Tris/EDTA buffer pH 9.0, blocked, and incubated with primary antibodies overnight at 4°C. The following primary antibodies were used: rabbit anti CD163 ([1:500] Abcam), mouse anti-albumin ([1:500] Sigma-Aldrich), and rabbit anti CD68 ([1:100] Cell Signaling Technology). Vehicle control tissue was used to assess non-specific antibody staining within the tissue constructs and a secondary fluorophore-conjugated antibody control was also performed on successive tissue sections as a procedural control. Alexa Fluor 488 or 594 secondary antibodies (ThermoFisher Scientific) were used where appropriate. Slides were visualized and imaged using Zeiss Axioskop microscope. Images were acquired with a Zeiss Axiocam IC camera and ZEN 2 (Blue Edition) software version 2.0.

### Image analysis

Five transverse tissue sections taken from the mid region of the tissue were used to assess the extent of collagen deposition within replicate tissues. The percent area collagen relative to the cross-sectional tissue area was quantified in representative tissue sections using ImageJ software and averaged across replicate tissues (n = 2–3 tissues per treatment group) [18]. Briefly, the image threshold was defined (*i*.*e*., Hue = 120/180; Saturation = 0/255; and Brightness = 140/230) to highlight all collagen positive areas within a section (blue). The region of interest was then demarcated by tracing the outer perimeter of the tissue. A binary image was then generated using the pre-defined threshold and the percent collagen positive area relative to tissue cross-sectional area was quantified for each tissue section. Image analysis data have been deposited in the Dryad Digital Repository and can be downloaded at doi: 10.5061/dryad.5rc1973.

### Cytokine measurements

The levels of cytokines released into the medium on treatment days 13 and 27 were assayed on the MESO QuickPlex SQ 120 Instrument using the Meso Scale Discovery V-PLEX Human Proinflammatory Panel 1 kit according to the manufacturer’s instructions. Samples and standards were prepared with minor modifications as described previously [9]. Only cytokines previously shown to be involved in modulating fibrogenic processes were analyzed [19]. Cytokine data have been deposited in the Dryad Digital Repository and can be downloaded at doi: 10.5061/dryad.5rc1973.

### RNA isolation

Tissue lysates were prepared for each treatment group by homogenization in TRIzol Reagent (ThermoFisher Scientific) using PreCellys RNase-free microfuge tubes and the Precellys 24 homogenizing instrument (Bertin Corp.). Total RNA was isolated using the Direct-Zol RNA MiniPrep kit per the manufacturer’s instructions (Zymo Research). RNA purity and yield were assessed using the NanoDrop 1000, version 3.5.2 (ThermoFisher Scientific). RNA integrity was assessed using the Agilent 2200 Tape Station System (Agilent Technologies). Optimal purity and minimum degradation for subsequent microarray analysis were defined by: A260/280 >1.8, A230/280 > 1.8, and RNA Integrity Number (RIN) > 8.0. Samples that did not meet the minimum concentration requirements (*i*.*e*., RNA concentration <33 ng/mL) were concentrated using a Savant SpeedVac Concentrator (ThermoFisher). Whole genome microarray profiling was performed by the UNC Functional Genomic Core facility on total RNA isolated from vehicle-, 10 ng/mL TGF-β1- and 0.209 μM MTX-treated tissues sampled at treatment days 14 and 28. For microarrays, sense-strand cDNA (ss-cDNA) was synthesized from 100 ng of total RNA using the GeneChip WT PLUS Reagent Kit (Affymetrix). Following purification, 5.5 μg of ss-cDNA were fragmented and end labeled with biotin before hybridizing to the array plate. The Affymetrix Clariom S HT Human 96-peg array was used with the Affymetrix Gene Titan Multi-Channel system. GeneTitan robotic instrumentation was used to perform the hybridization, washing, and scanning of the peg array using the Affymetrix GeneTitan Hybridization, Wash, and Stain Kit for WT Arrays.

### Gene expression analysis

Data summarization and QC was performed using the Transcriptome Analysis Console (TAC) software version 4.0 (ThermoFisher). Affymetrix CEL files (n = 36) were normalized using the Robust Multi-array Average method with a log base 2 (log2) transformation [20]. All subsequent analyses were performed using Partek Genomics Suite version 6.6. Principal component analysis (PCA) was used to evaluate the overall performance of the arrays and identify outliers. Samples > 3 SD away from the mean were omitted from analysis. A filtering step was performed to remove low expression probe sets. A three-way analysis of variance (ANOVA) was performed with experimental factors (i.e., treatment, time, +/-KCs) and interaction terms (i.e., time × treatment, treatment × +/-KCs, +/-KCs × time, and treatment × time × KCs). Differential expression for each condition was determined by linear contrasts. Probability values were adjusted for multiple comparisons using a false discovery rate of 5% (FDR = 0.05) [21] and an absolute value fold change (FC) cutoff of > 1.5 was also used as noted below. Pathways and toxicity lists enriched among statistically significant, differentially expressed genes in the data were identified using the Tox Analysis module in Ingenuity Pathway Analysis (Ingenuity Systems; Build version 460209M; Content version: 39480507). Gene expression data generated for this manuscript can be downloaded in its entirety from the Gene Expression Omnibus repository under the accession number GSE113630. All data are MIAME compliant.

### Other statistical analyses

Statistical analyses for all other endpoints were performed using GraphPad Prism statistical software version 7.01. Unless otherwise noted, results are expressed as the mean of n = 5–7 tissue replicates + standard error of the mean (SEM). Statistical significance of treatment-induced differences relative to vehicle-treated control was determined using a two-way ANOVA where appropriate, with post hoc Sidak’s multiple comparisons test. Outliers were identified using Grubbs’ test (α = 0.05).

## Results

### The incorporation of Kupffer cells shortens the general injury window observed with extended compound exposure

#### Standard tissue model (-Kupffer cells)

**Fig 1:**
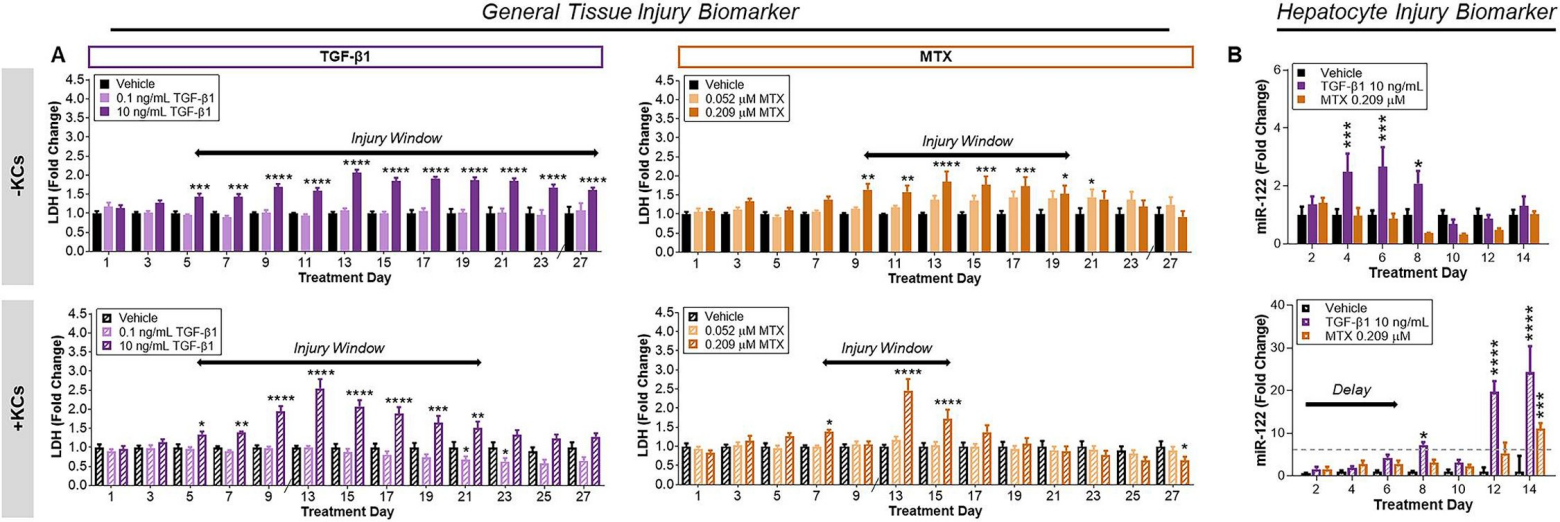
Temporal profile of injury markers with repeated compound exposure. (A) LDH release during the entire treatment time course as an indicator of general tissue injury for the standard (-KCs) and modified (+KCs) tissue model. (B) Abundance of miR-122 in cell culture medium as a hepatocyte specific injury marker during the first 14 days of treatment for each model. Concentrations of TGF-β1 and MTX in which an LDH response was observed are represented. Data represent the mean of the fold change + SEM (n = 5–7). Significance determined using two-way ANOVA with post hoc Dunnett’s multiple comparisons. *P < 0.05, **P < 0.01, ***P < 0.001, ****P < 0.0001.

LDH release in spent tissue culture medium was used as a biomarker of generalized tissue injury throughout the course of treatment. During this period, LDH release gradually declined and approached steady-state levels by treatment day 7 with minor differences in the rate of decline across the various treatments (S3A Fig). Evaluation of LDH release over an extended period of time showed that the highest concentration of each agent tested resulted in a significant increase in LDH relative to vehicle-treated control (Fig 1A). Treatment with 10 ng/mL TGF-β1 resulted in a 1.5 to 2.0-fold increase in LDH which was sustained throughout the exposure period. A 1.5 to 2.0-fold increase in LDH spanned treatment days 9–19 for tissues treated with 0.209 μM MTX with a gradual decrease in LDH release continuing until treatment day 27. For the lower concentration of each agent there was no statistically significant increase in LDH observed over the treatment time course apart from 0.052 μM MTX on treatment day 21.

While LDH has been traditionally used as an indicator of cytotoxic responses in monocultured cells, it only provides a broad measure of the injury response across all the cell types in the bioprinted model [22]. Given the complex nature of the fibrogenic response and strong association with hepatocyte injury, we also measured miR-122 in the culture medium as a more sensitive and specific biomarker of hepatocyte injury in response to the highest concentrations of both agents [22]. Similar to LDH release, a decline in miR-122 was observed across all treatments over time (S3B Fig) Statistically significant elevations in miR-122 relative to vehicle-treated control was evident during the first two weeks of the treatment period for 10 ng/mL TGF-β1 treatment group, beginning on treatment day 4 (Fig 1B). No elevation in miR-122 was observed with 0.209 μM MTX during the initial 14 days of treatment (Fig 1B).

#### Modified tissue model (+Kupffer cells)

A gradual decline in LDH release was also observed during the initial 9 days of exposure for all treatments in the modified tissue model (S3A Fig). Relative to vehicle control, repeated exposure to the highest concentration of each fibrogenic agent resulted in a monophasic increase in LDH by the mid treatment time point (Fig 1A). Treatment with 10 ng/mL TGF-β1 resulted in a 1.5 to 2.5-fold increase in LDH which spanned treatment days 5–21 while treatment with 0.209 μM MTX resulted in a 1.5 to 2.5-fold increase in LDH which spanned treatment days 7–15. Compared to the standard tissue model responses, the window of significant treatment-induced LDH release was shortened for both agents when KCs were incorporated into the model. As seen with the standard model, there were no statistically significant increases in LDH observed at the lower concentrations of each agent. Although subsequent experiments were performed at both concentrations, analysis in the present study focused on the higher concentrations of TGF-β1 (10 ng/ml) and MTX (0.209 μM) where an LDH response was observed in both tissue models.

The abundance of miR-122 in the culture medium also declined during the initial exposure period for all treatments in the modified tissue model (S3B Fig). Within the same timeframe, significant differences relative to vehicle control were observed for both agents (Fig 1B). Treatment with 10 ng/mL TGF-β1 resulted in elevations in miR-122 release beginning on treatment day 8 and peaking at 20-25-fold on days 12–14 (Fig 1B). An approximately 10-fold increase in miR-122 was observed in response to 0.209 μM MTX on treatment day 14. In comparison to the standard tissue model response, the significant increase in miR-122 within the first 14-days of treatment was delayed following treatment with TGF-β1. Treatment with MTX only elicited a significant miR-122 response when KCs were incorporated.

### Incorporation of Kupffer cells does not significantly perturb tissue function following extended TGF-β1 and methotrexate treatment

#### Standard tissue model (-Kupffer cells)

**Fig 2:**
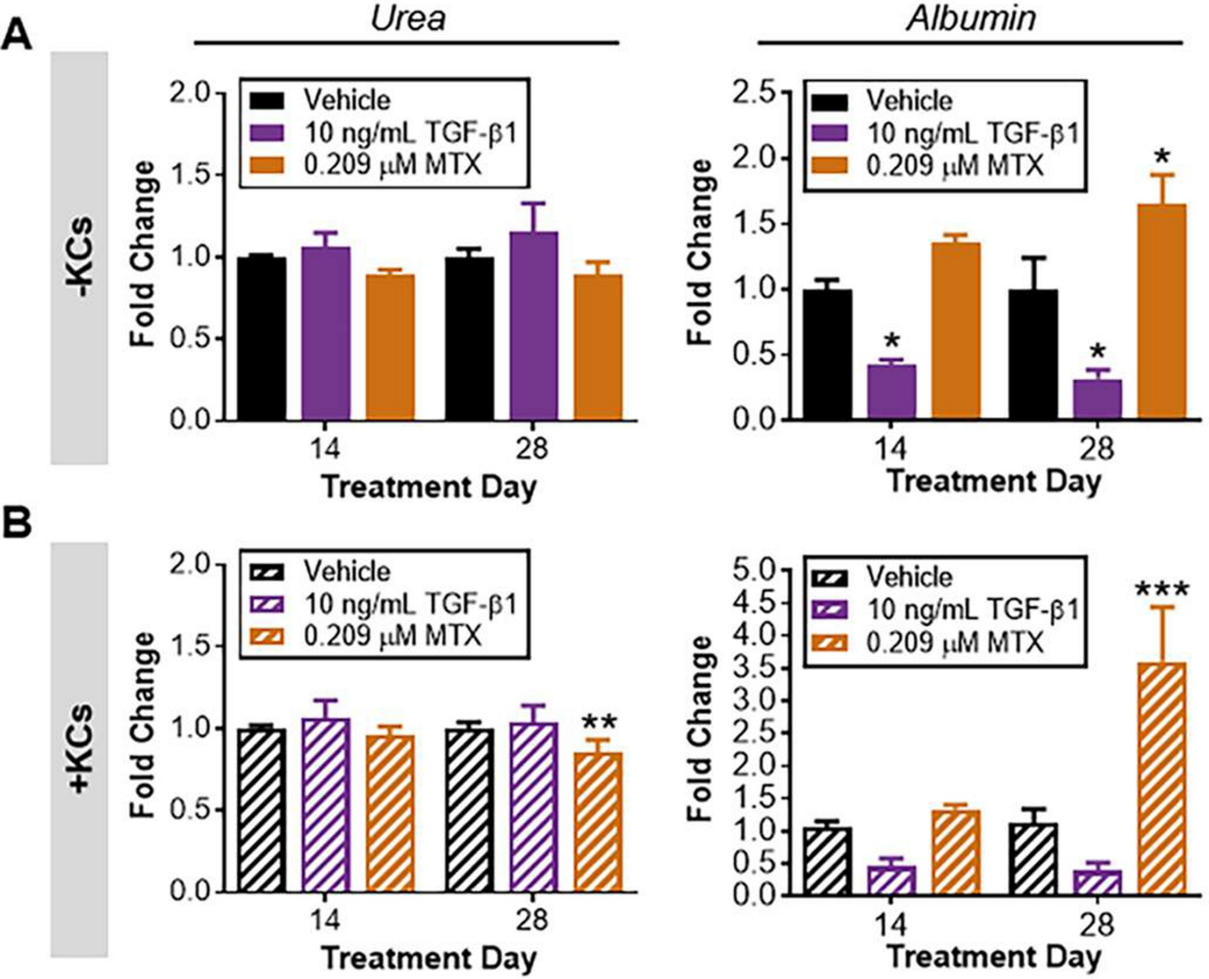
Biochemical markers of hepatocyte function at treatment day 14 and 28. Urea and albumin were measured in spent cell culture medium for each treatment group to benchmark injury profiles over time in the (A) standard tissue model and (B) modified tissue model. Data represent the mean of the fold change + SEM (n = 5–7). Significance determined using two-way ANOVA with post hoc Sidak’s multiple comparisons, *P < 0.05, **P < 0.01, ***P < 0.001.

Urea and albumin were assessed as markers of hepatocyte function in response to the highest concentration of fibrogenic agents at treatment days 14 and 28 (Fig 2A). In the standard tissue model, urea production was unchanged following treatment with TGF-β1 and MTX at each timepoint of exposure. Albumin production was reduced by approximately half in response to TGF-β1 at both timepoints but increased in response to MTX at treatment day 28 relative to vehicle-treated control (Fig 2A).

#### Modified tissue model (+Kupffer cells)

In the modified tissue model, urea output also remained comparable to vehicle-treated controls following TGF-β1 exposure at both the 14-day and 28-day time points (Fig 2B). However, treatment with MTX resulted in a significant decline in urea production by treatment day 28. Albumin production was also reduced by approximately half on both treatment days for tissues treated with TGF-β1, although this decline was not statistically significant. Similar to the standard tissue model, a 3.6-fold increase in albumin output relative to vehicle control was observed in response to MTX on treatment day 28.

### Baseline differences in collagen deposition observed in Kupffer cell containing tissues

#### Standard tissue model (-Kupffer cells)

**Fig 3:**
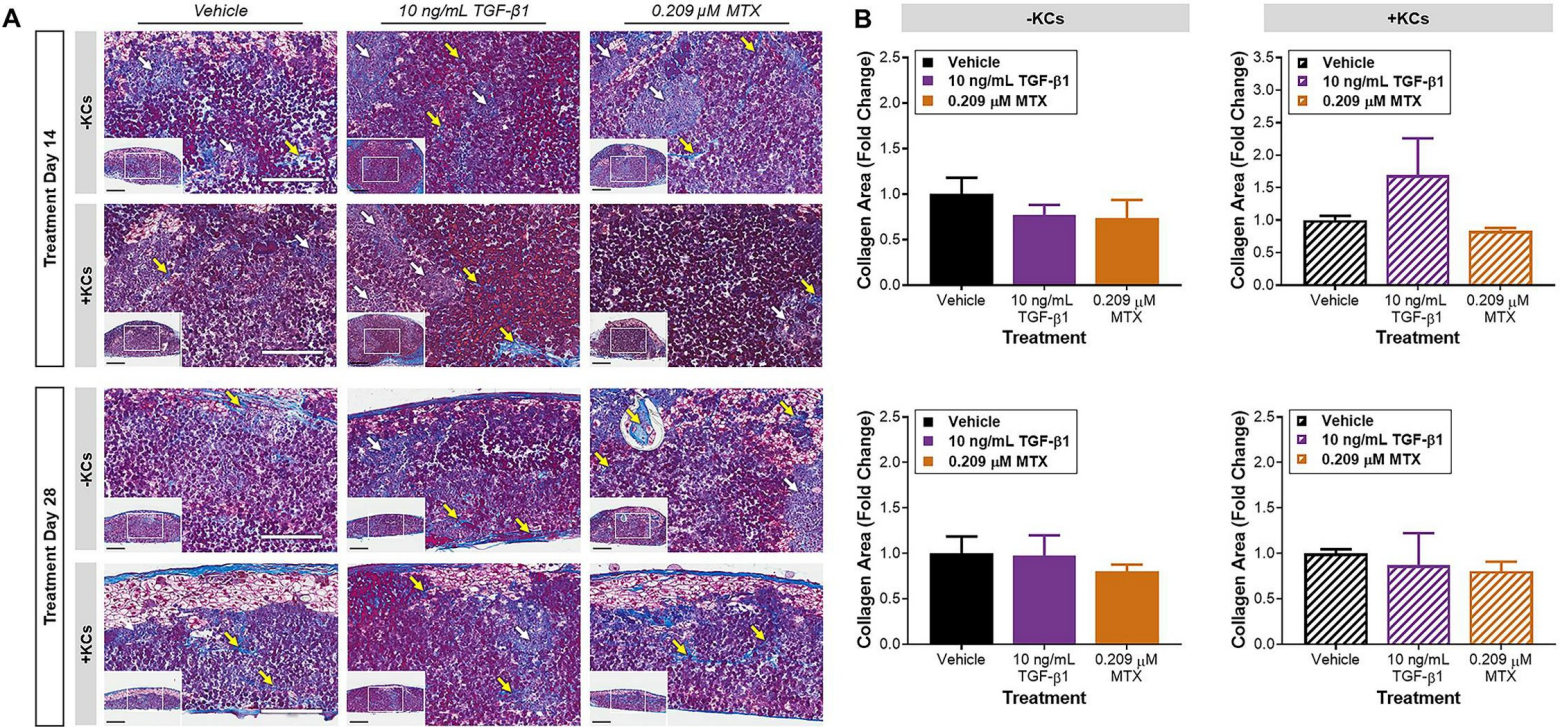
Collagen deposition in tissue sections. (A) Representative photomicrographs of Gomori’s trichrome-stained tissue sections at treatment day 14 and 28. White arrows denote diffuse areas of collagen deposition and yellow arrows denote thicker collagen fibers. Inset indicates area used for quantification. Scale bar = 300 **μ**m, inset scale bar = 75 **μ**m. (B) Fold change collagen relative to model-matched, vehicle-treated control. Data represent the mean of the fold change + SEM at each timepoint (n = 2).

Gomori’s trichome staining was used to examine collagen deposition in tissues where there was an observed shortening of the treatment-induced injury window with the incorporation of KCs (Fig 3A). In the standard model, collagen deposition patterns were mostly diffuse throughout the tissue originating from nodules corresponding to the non-parenchymal compartment (white arrows). Areas of brilliant blue staining (yellow arrows) were also observed indicating organized bands of collagen fibers bridging areas of more diffuse collagen staining. Collagen deposition resulting from TGF-β1 and MTX treatments were mainly localized to the periphery of the tissue resulting in consolidation and encapsulation of the tissue with collagen positive (blue) fibrous bands. Areas of collagen staining (insets, Fig 3A) were quantified in two tissues from each treatment. However, no significant differences in collagen deposition were observed relative to time-matched, vehicle control in the small number of tissues evaluated (Fig 3B).

#### Modified tissue model (+Kupffer cells)

The inclusion of KCs did not significantly alter tissue responses to either TGF-β1 or MTX treatment (Fig 3). However, regardless of treatment, a general decrease in tissue mass was observed by day 28 (insets, Fig 3A). Interestingly, comparison of the percent area of collagen in the modified versus standard tissue model demonstrated a trend of decreased collagen positive areas with the incorporation of KCs at treatment day 14 (S4 Fig.). Ballooning, degenerating hepatocytes appeared to be more prevalent in the apical region of the modified tissue model relative to the standard tissue model at treatment day 28 (Fig 3A). By this later treatment time point, collagen deposition within the center of the tissues was comparable across tissue compositions (S4 Fig.).

### Kupffer cells alter cytokine responses in bioprinted liver tissues exhibiting mild injury

**Fig 4:**
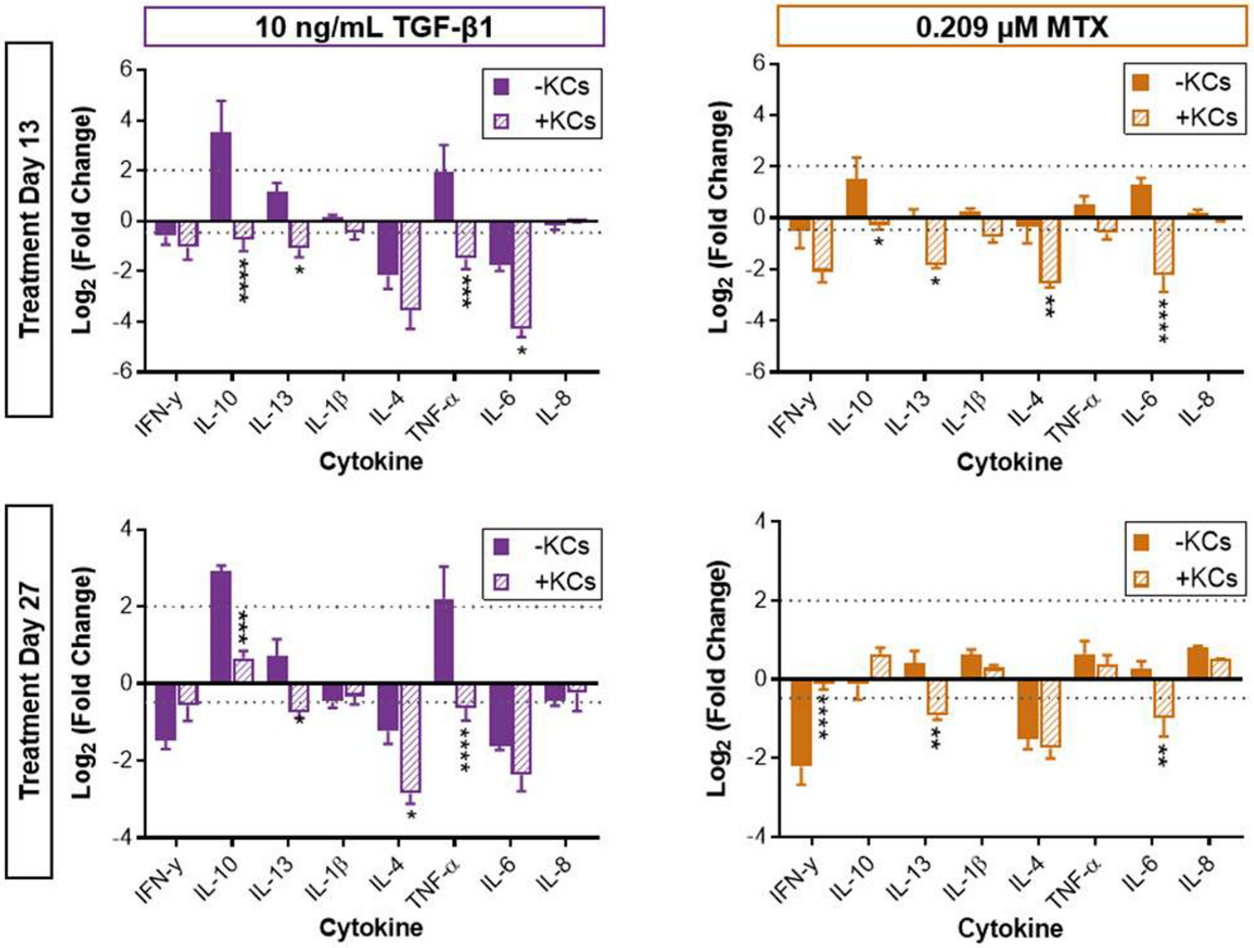
Abundance of inflammatory cytokines in the culture medium at treatment day 13 and 27. Impact of treatment on cytokine profiles across tissue models for each timepoint. Data represent the mean Log_2_ of the fold change relative to tissue-matched vehicle-treated control mean + SEM (n = 5). Significance determined using two-way ANOVA with post hoc Dunnett’s multiple comparisons. *P < 0.05, **P < 0.01, ***P < 0.001, ****P < 0.0001. KCs had a significant impact on cytokine profiles at treatment day 13 for both treatments and at treatment day 27 for TGF-β1 treatment (*P* < 0.0001, two-way ANOVA).

A panel of cytokines was measured in spent medium samples at treatment days 13 and 27 to assess the impact of KCs on the treatment-induced inflammatory response. The addition of KCs resulted in an overall higher cytokine abundance measured in the vehicle-treated control tissues that was significant on treatment day 13 (S5 Fig). However, the incorporation of KCs resulted in a global decrease in treatment-induced fold change in inflammatory cytokine production compared to the standard model at treatment day 13 for both TGF-β1 and MTX (Fig 4). Cytokines that were significantly different between tissue compositions included IL-10, IL-13, IL-4, TNF-α, and IL-6. At treatment day 27, the observed trends in cytokine production following treatment were comparable to day 13 tissues regardless of tissue composition for both TGF-β1 and MTX (Fig 4). Because tissues were cultured over an extended period of time, the presence of KCs within tissues at each time point was also confirmed with CD68 immunohistochemistry (S6 Fig).

### Impact of cytokine- versus drug-mediated gene expression suggests a context-dependent role for Kupffer cells

**Fig 5:**
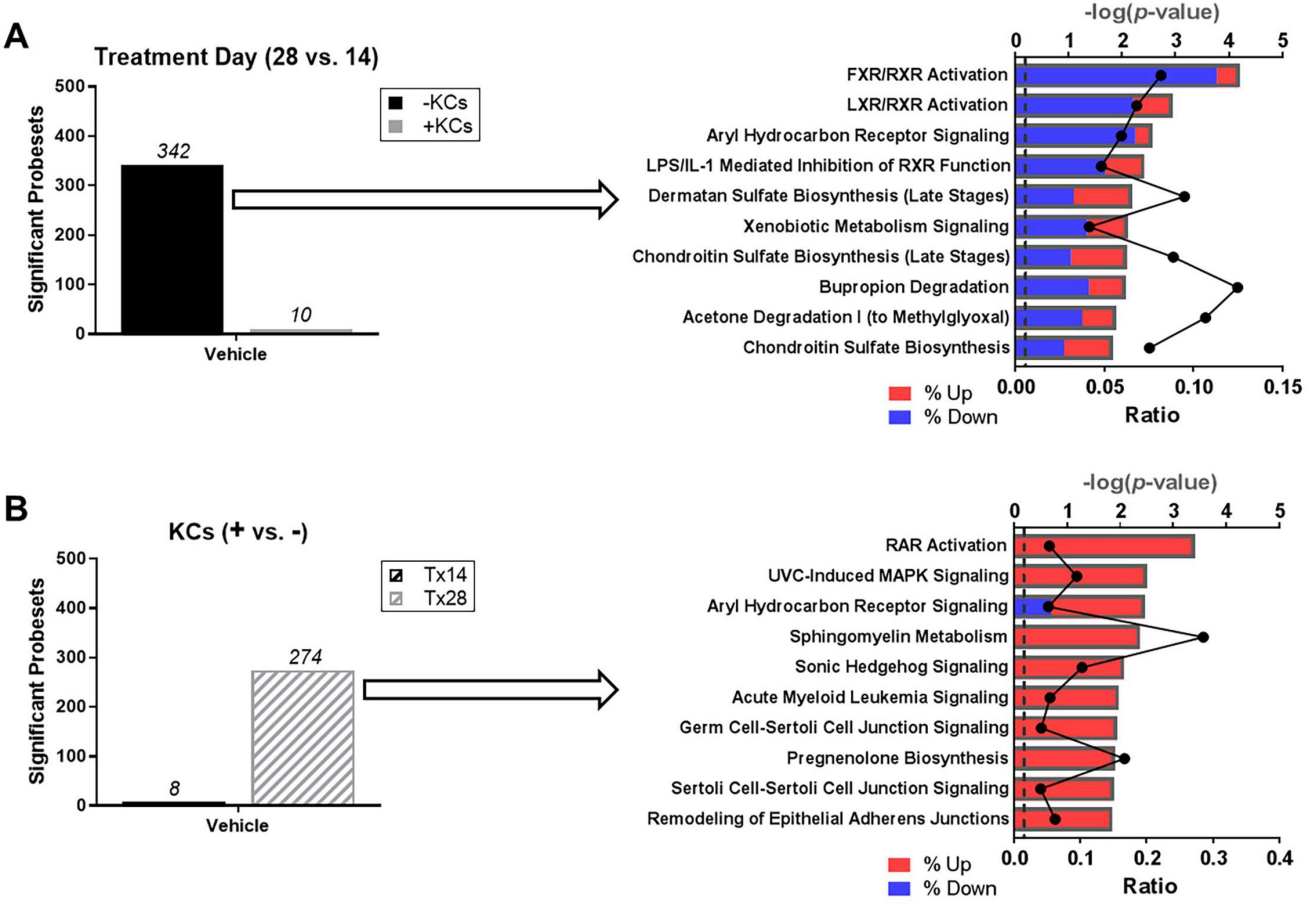
Impact of time and Kupffer cells on baseline tissue gene expression profiles. (A) Number of differentially expressed genes that vary over time in the standard and modified tissue model treated with vehicle. Enriched pathways from the list of 342 differentially expressed genes observed in the standard tissue model. (B) Number of differentially expressed genes that vary with the incorporation of Kupffer cells in vehicle-treated tissues at treatment day 14 and 28. Enriched pathways from the list of 274 differentially expressed genes observed at treatment day 28. Data represent the -log(*P*-value) for pathway significance (bars) and the ratio (black line) of the number of genes from the dataset divided by the total number of genes comprising a pathway from the IPA knowledge base. The shading within each bar represents the proportion of down- (blue) versus up-regulated (red) genes within a particular pathway. Abbreviations: FXR, farnesoid X receptor; LPS, lipopolysaccharide; LXR, liver X receptor; MAPK, mitogen activated protein kinase; RAR, retinoid acid receptor; RXR, retinoid X receptor; UVC, ultraviolet C.

Microarray profiling was performed to gain insight into the progressive series of events that occur with TGF-β1 and MTX treatment and the impact of KCs on the response profiles of each agent. Because exposures were conducted over an extended period of time, the impact of time on baseline gene expression was first assessed for both the standard and modified tissue model (Fig 5A). Comparing treatment day 28 versus treatment day 14, 342 genes were differentially expressed in the standard model, whereas only 10 genes were differentially expressed in the KC-modified model. Pathways enriched among the 342 significantly differentially expressed genes in the standard model corresponded to homeostatic mechanisms including lipid, glucose, cholesterol, bile acid, and xenobiotic metabolism. Within each pathway, the majority of genes were downregulated on day 28 versus 14. Next, we compared the modified and standard tissue model gene expression profiles to assess the impact of KCs at each timepoint (Fig 5B). At 14 days, 8 genes were differentially expressed across the tissue models versus 274 genes by culture day 28. Pathways enriched among these 274 significant, differentially expressed genes in the modified tissue model included signaling for cellular growth/proliferation, cell movement/development, cell-cell, and cell-matrix interactions. Within each pathway, the majority of genes were upregulated when KCs were incorporated into the model.

**Fig 6:**
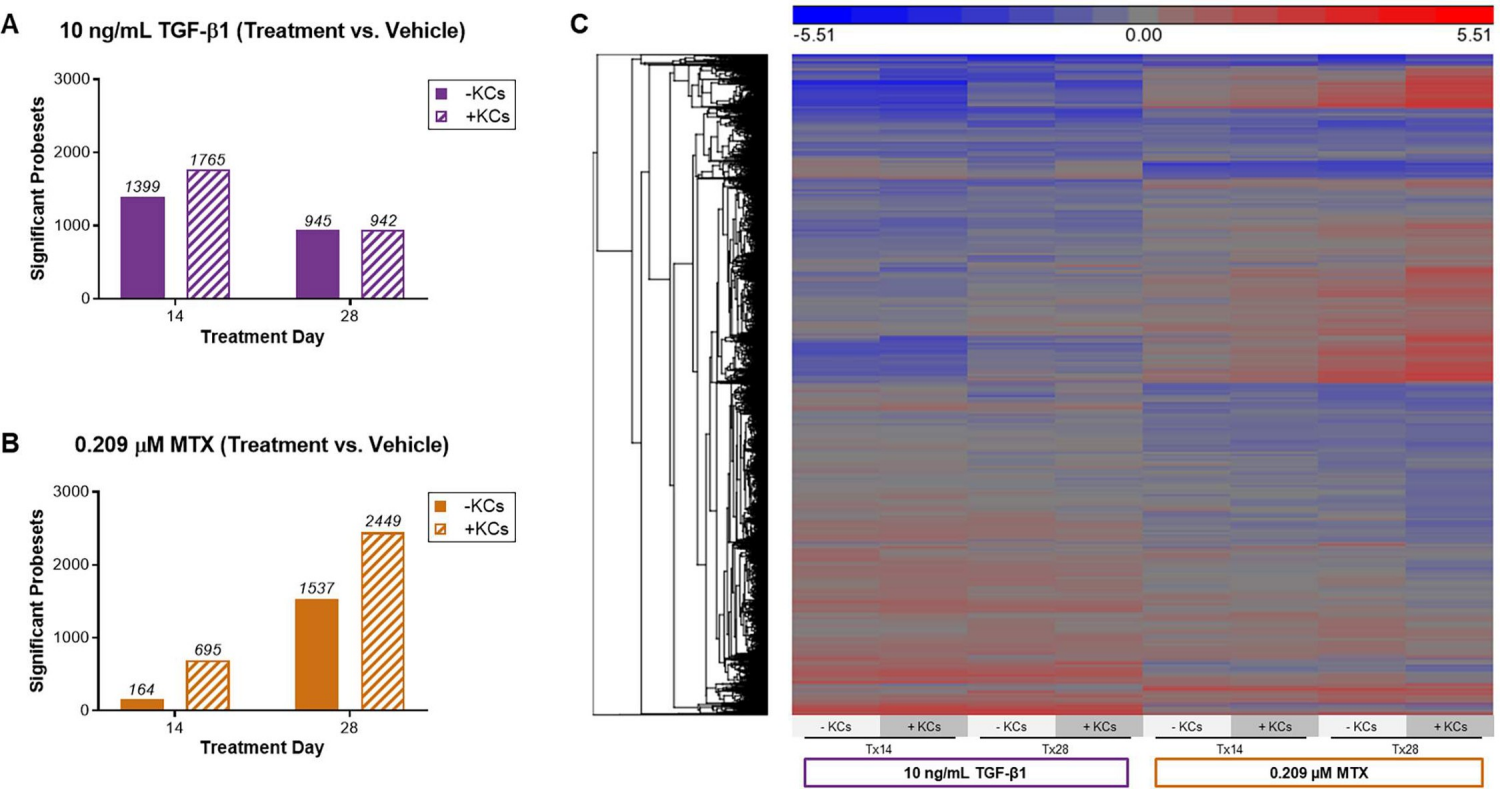
Impact of treatment on tissue gene expression profiles under various conditions. (A) Number of differentially expressed genes that vary over time in the standard and modified tissue model treated with 10 ng/mL TGF-β1. (B) Number of differentially expressed genes that vary over time in the standard and modified tissue model treated with 0.209 μM MTX at treatment day 14 and 28. (C) Hierarchical clustering of all significant probe sets as a function of treatment, model composition, and exposure time. Differential expression was determined by linear contrasts between each treatment and the vehicle-treated control for each model and timepoint of exposure (False Discovery Rate *P* < 0.05 and |Fold Change| > 1.5). The shading reflects the directionality (red, upregulated and blue, downregulated) and magnitude (darker, larger fold change) of the Log_2_ (ratio) for the contrast.

Treatment-related effects relative to vehicle control were assessed in both tissue models at each timepoint. Fig 6A and 6B summarize the number of significant, differentially expressed genes in response to each treatment condition. At treatment day 14, the number of significant, differentially expressed genes in response to TGF-β1 was increased for the modified tissue model, whereas the number was similar across models following 28 days of TGF-β1 treatment. Treatment with MTX resulted in an overall increase in the number of differentially expressed genes at both timepoints in the modified tissue model compared to the standard tissue model (Fig 6B). The heat map illustrates the differences in gene expression profiles over time and across tissue compositions for each treatment and summarizes the variance observed following exposure to TGF-β1 versus MTX (Fig 6C).

**Fig 7:**
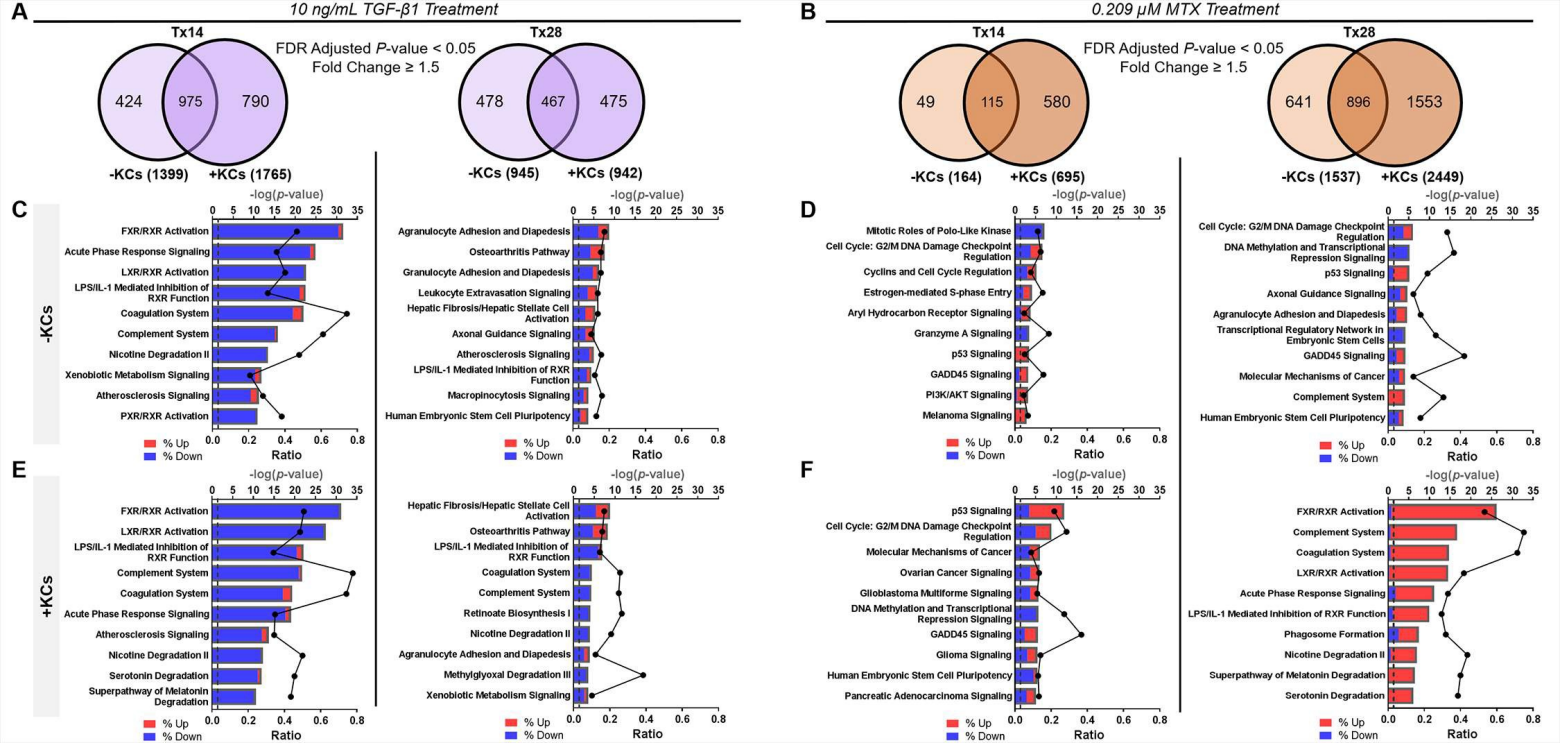
Summary of differentially expressed genes and enriched canonical pathways for each treatment. (A,B) Venn diagram of differentially expressed genes in TGF-β1 and MTX-treated tissues relative to vehicle-treated control tissues for the standard (-KCs) versus modified (+KCs) tissue model at treatment day 14 and 28. (C,D) Enriched pathways from the list of differentially expressed genes observed in the standard tissue model for each treatment. (E,F) Enriched pathways from the list of differentially expressed genes observed in the modified tissue model for each treatment. Data represent the -log(*P*-value) for pathway significance (bars) and the ratio (black line) of the number of genes from the dataset divided by the total number of genes comprising a pathway from the IPA knowledge base. The shading within each bar represents the proportion of down (blue) versus upregulated (red) genes within a particular pathway. Abbreviations: AKT, serine-threonine protein kinase; FDR, false discovery rate; FXR, farnesoid X receptor; GADD45, growth arrest and DNA damage; LPS, lipopolysaccharide; LXR, liver X receptor; PI3K, phosphoinositide 3-kinase; PXR, pregnane X receptor; RXR, retinoid X receptor.

Of the genes that were differentially expressed in response to TGF-β1, there was strong overlap in the number of genes in the standard versus modified tissue model at both treatment timepoints (Fig 7A). Among genes that were differentially expressed at treatment day 14, cell movement, cell-cell interactions, injury, and inflammatory response pathways were enriched regardless of whether KCs were present in the tissue (Fig 7C and 7E). At treatment day 28, the top 10 pathways enriched among differentially expressed genes mainly converged on cell death/survival, cell-cell interaction, cellular function/maintenance, and hepatic fibrogenesis (Fig 7C and 7E). While these pathways were generally conserved, the ranked order was different across tissue compositions.

Following MTX treatment, the number of differentially expressed genes at treatment day 14 and 28 exhibited less overlap between the two tissue models (Fig 7B). While the top enriched pathways were mainly involved in DNA replication/repair and cell cycle signaling in the standard tissue model, the pathways enriched in the modified tissue model represented more complex signaling events involving cellular crosstalk (Fig 7D and 7F). MTX treatment of the standard tissue model at 28 days resulted in the enrichment of pathways involved in cell migration, cell cycle, cell death and survival (Fig 7D). In the modified tissue model, 28-day MTX treatment resulted in the enrichment of pathways involved in cell death, survival, maintenance of cellular function, and inflammation (Fig 7F).

## Discussion

According to the proposed Adverse Outcome Pathway (AOP) for Liver Fibrosis, initial hepatocyte injury leads to the activation of KCs and HSCs which ultimately leads to collagen accumulation [23, 24]. TGF-β1, a potent profibrogenic cytokine, can also cause direct HSC activation and collagen deposition [23, 24]. Given the complex and dynamic nature of the response, the ability to dissect the early response at the organ level during injury requires an *in vitro* model that is able to recapitulate basic fibrogenic features and incorporates cell types relevant to the response [23, 24]. Here we describe the continued development of a 3D bioprinted liver tissue model for studying progressive liver fibrosis by assessing the impact of KCs in modulating the early response to TGF-β1- and MTX-induced fibrotic injury. The flexibility of incorporating specific cell types in a tissue-relevant context has been a significant innovation in designing physiologically relevant platforms with which to understand basic fibrogenic mechanisms and also gain insight into the impact of other cell types in modulating this outcome [9]. As such, this model is uniquely suited to provide a better understanding of how KCs modulate the early response to fibrogenic agents in a model of progressive tissue injury and fibrogenesis [9, 13].

As previously observed, a significant post-print acclimation period (7–10 days) was required for injury biomarkers to reach steady-state levels, which was highly correlated with proinflammatory cytokines in the standard tissue model [9]. This “wound healing response” may be due to the activation of HSCs upon mechanical stimulation (*i*.*e*. printing), which appear to re-establish a quiescent-like phenotype over time [9]. Assessment of additional injury biomarkers in the current study further confirm that future studies may benefit from initiating treatment regimens to assess drug-related effects on fibrogenic processes after this initial acclimation phase. Because both tissues with and without KCs remained viable for 28 days, chronic toxicity studies can still be performed. This is particularly important because fibrosis manifests over the course of continued mild injury. While extended treatment with fibrogenic agents resulted in a sustained mild injury response for both treatments, a shortening of the general injury profile (LDH release) was observed when KCs were incorporated in the model. Interestingly, peak LDH release was slightly higher in tissues containing KCs (~2.5-fold) compared to the standard tissue model (~2.0-fold). Additional experiments would be required to determine if these differences are meaningful.

In accordance with the proposed AOP for Liver Fibrosis [23, 24], our data with miR122 suggest early hepatocyte injury precedes general, sustained tissue injury in the standard tissue model for TGF-β1. However, the apparent delay in miR-122 release observed in the modified tissue model with TGF-β1 treatment implicates a potential role for KCs in the attenuation of early hepatocyte injury. The fold increase in miR-122 was also much greater during extended TGF-β1 exposure. This suggests that while KCs may attenuate hepatocyte injury response during the initial exposure timeframe, continued exposure to fibrogenic agents might result in an exacerbation of the response. Continued exposure to MTX in the modified tissue model also resulted in a delayed, but significant elevation in miR-122 release which was not observed in the standard tissue model. The lack of elevated miR-122 in the standard model was surprising but may suggest only mild hepatocyte stress occurring at the concentrations of MTX used here.

Trends in urea and albumin output support that the observed elevations/patterns of LDH and miR-122 reflected only mild injury to the hepatocytes. While urea production remained largely unchanged with TGF-β1 and MTX, the impact of treatment on albumin synthesis was apparent for both treatments. Decreased albumin output following TGF-β1 treatment was concordant with the observed LDH and miR-122 responses. However, because urea production was not significantly altered with treatment, this suggests hepatocytes within the tissues retained aggregate functional capacity. The significant increase in albumin output following MTX treatment in the modified tissue model is consistent with previous results and is likely reflective of adaptive processes (*e*.*g*., activation of NPCs and secretion of factors to support the synthesis of albumin) or changes in the release of intra-tissue albumin with mild injury [9]. However, the exact mechanisms underlying this response and impact on fibrogenic outcome warrant further investigation.

The lower concentrations pursued in the current study resulted in a more gradual accumulation of collagen within the tissues over an extended exposure timeframe. This exposure scenario was employed to emulate chronic injury leading to fibrogenesis given our interests in the initiating and adaptive events underlying the response [9]. Despite the lack of significant collagen deposition with TGF-β1 and MTX treatment as observed in previous studies, the incorporation of KCs resulted in less collagen overall at the mid treatment timepoint.

No difference was observed in the standard tissue model following TGF-β1 and MTX treatment at each timepoint. In the modified tissue model, KCs appeared to impact the TGF-β1 response at treatment day 14 versus 28 with no significant change following MTX treatment. Although LDH release was reduced (*i*.*e*., shortening of the injury window), hepatocyte injury as indicated by miR-122 was increased before the 28-day timepoint. This data is concordant with the evidence of collagen deposition in the tissues by treatment day 28. However, the potential shift in the incorporated KC population over time or perturbations in functionality could lead to the observed decrease in cellularity and/or increase in collagen deposition within bioprinted liver tissues following extended compound exposure. It is also important to consider that the quantitative detection of collagen deposition/modulation by KCs resulting from treatment, may require a much larger sample size and additional analytical methods.

The global dampening of compound-induced cytokine secretion at treatment day 13 in the KC-modified model was consistent with histological outcomes. This dampening is consistent with the observed shortening of the general injury window (LDH) but somewhat surprising in view of the increased hepatocyte injury (miR-122). Although cytokines known to drive wound healing were significantly decreased following treatment in the modified tissue model, reduction in certain anti-inflammatory cytokines such as IL-10 exhibited disparate trends than anticipated in the context of a reduced injury window [19, 25]. Because cytokines were measured in the medium at the mid treatment time point near or at the apex of the injury window for each model, it is plausible that transient changes in cytokine production prior to the detection of significant differences in LDH release could have exhibited a more immunomodulatory profile.

While the presence of KCs was confirmed at later time points, additional studies are required to understand the dynamics of the KC population within the tissues over time particularly with treatment. Although CD68 is a common marker to detect macrophages within tissue sections, the ability to discern macrophage polarization based on cell surface markers alone remain a challenge [26]. However, given the unique environment in which the recruitment of extrahepatic monocytes is not considered in this system, bioprinted liver tissues could also provide an important insight into the phenotypic heterogeneity of KCs isolated from perfused liver and their role in modulating injury and fibrogenesis. Furthermore, extrahepatic immune cells could also be incorporated in future versions of the model to tease apart resident versus recruited cell-mediated fibrogenic processes during compound-induced liver injury.

**Fig 8:**
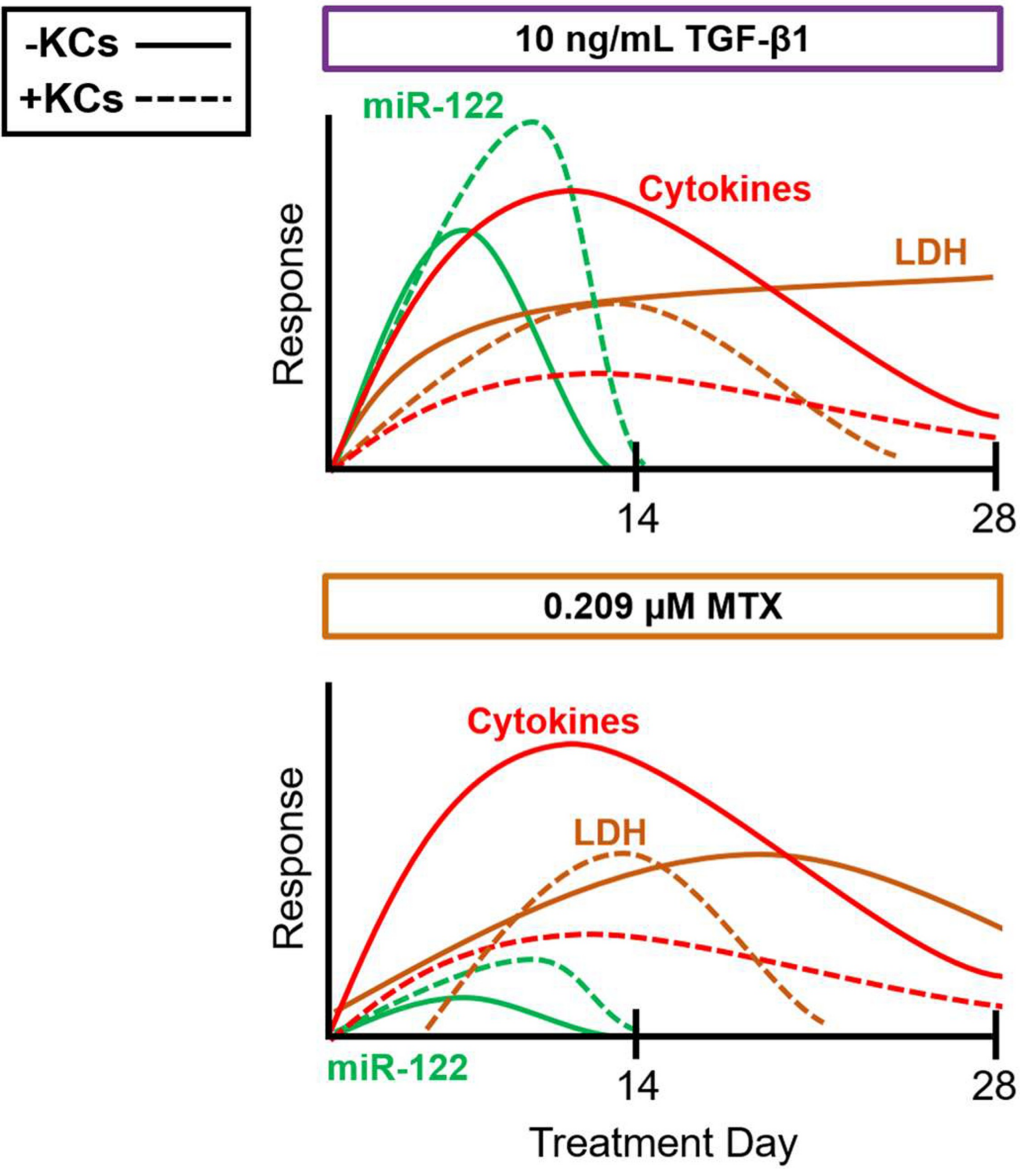
Summary of biochemical endpoints with treatment. Conceptual diagram depicting the trends in biochemical endpoints over time with treatment.

Although treatment with TGF-β1 and MTX resulted in similar biochemical response profiles over an extended treatment period (summarized in Fig 8) the differential impact of each agent was reflected in the gene expression profiles over time for each tissue composition. Overall, the conservation of pathways observed in tissues treated with TGF-β1 regardless of tissue composition and time could be a result of the progression of the response. Alternatively, this could also be reflective of treatments which impact different nodes within the AOP framework [23, 24]. The TGF-β1 response could be explained by the proposed AOP framework in which its effects as a potent fibrogenic cytokine, act in the intermediate steps of the response (*i*.*e*., after hepatocyte injury) [23, 24]. However, the fibrogenic response evoked by MTX treatment may act mainly via injury to hepatocytes which is proposed to precede the downstream fibrogenic response.

While MTX evoked a very mild tissue injury response over time, the presence of KCs clearly modulated the injury response as evidenced by differential enrichment of key pathways involved in cell death, growth arrest, and complex signaling events between various cell types. In the modified tissue model, the representation of pathways at the later phase of the response was most similar to the enriched set of pathways observed for the modified tissue model following treatment with TGF-β1 at treatment day 14. This suggests that while compound-specific effects may be discerned early on, gene expression signatures become more alike over time with the progression of the response. Because the baseline gene expression changes and biochemical profiles were different in the standard versus modified tissue model, and the progression of fibrotic injury was different for TGF-β1 and MTX within the treatment timeframe, it is also important to benchmark the interpretation of the data with these findings.

Taken together, these results further demonstrate the important context-dependent role of KCs in the initial injury/fibrogenic response and progression over time. We also highlight the utility of more advanced organotypic models as an integrative approach to evaluate complex temporal responses underlying early compound-induced fibrotic injury. Understanding the role of resident KCs during these early fibrogenic events is not only important to assess the role of resident cell-derived inflammatory processes in the initial response but is also a fundamental aspect to consider in the development of relevant *in vitro* culture systems for fibrosis-associated compound risk and therapeutic assessment.

## Supporting information

S1 Fig Dose-response curves for MTX. Dose response curves were fit to LDH fold change values for MTX treatments on days (A) 7, (B) 9, and (C) 11. Average values across the 3 days were used to obtain LC20 (0.052 μM) and LC50 (0.209 μM) concentrations used to conduct the present studies. Data represent the mean of the fold change + SEM (n = 3–5) for each concentration.
(TIF)

S2 Fig General injury profiles across tissue prints during the first 14 days of treatment. (A) LDH response profiles for all treatment groups for the standard (-KCs) tissue model. (B) LDH response profiles for all treatment groups for the modified (+KC) tissue model. Data represent the mean of the fold change + SEM (n = 5–7) for each model. Significance determined using two-way ANOVA with post hoc Dunnett’s multiple comparisons. *P < 0.05, **P < 0.01, ***P < 0.001, ****P < 0.0001.
(TIF)

S3 Fig Raw LDH and miR-122 release over time. (A) LDH response profiles for 10 ng/mL TGF-β1 and 0.209 μM MTX for the standard (-KCs) and modified (+KCs) tissue model. (B) Corresponding miR-122 response profiles for each tissue model. Data represent the average raw vehicle values (black line) and average raw treatment values (colored lines) + SEM (n = 5–7).
(TIF)

S4 Fig Percent area collagen from trichrome-stained tissues. Data represent the mean of the fold change + SEM at each timepoint relative to effects observed in the standard tissue model (n = 2). At 14 days, KCs had a significant impact on collagen deposition (*P*< 0.01, two-way ANOVA).
(TIF)

S5 Fig Impact of Kupffer cells and time on baseline cytokine abundance. Box and whisker plots are used to describe the mean and range of the Log (Cytokine Concentration). Significance determined using two-way ANOVA with post hoc Sidak’s multiple comparisons. *P < 0.05 and ***P < 0.001. KCs had a significant impact on cytokine profiles at (A) treatment day 13 (*P* < 0.0001, two-way ANOVA) but not at (B) treatment day 27. Time had a significant impact on cytokine profiles in the (C) standard tissue model (*P* < 0.01, two-way ANOVA) and (D) modified tissue model (*P* < 0.001, two-way ANOVA).
(TIF)

S6 Fig CD68 immunohistochemistry at treatment days 14 and 28 for vehicle-treated tissue. Representative photomicrographs of vehicle-treated tissue for the standard (-KCs) and modified (+KCs) tissue model. CD68 (red) was used to label KCs within the tissues. Scale bar **=** 100 μm.
(TIF)
