# Supplementary figures and images for: Bioprinted liver provides early insight into the role of Kupffer cells in TGF-β1 and methotrexate-induced fibrogenesis
Source: PLoS One. 2019 Jan 2;14(1):e0208958. doi: 10.1371/journal.pone.0208958 (PMC6314567; doi:10.1371/journal.pone.0208958)

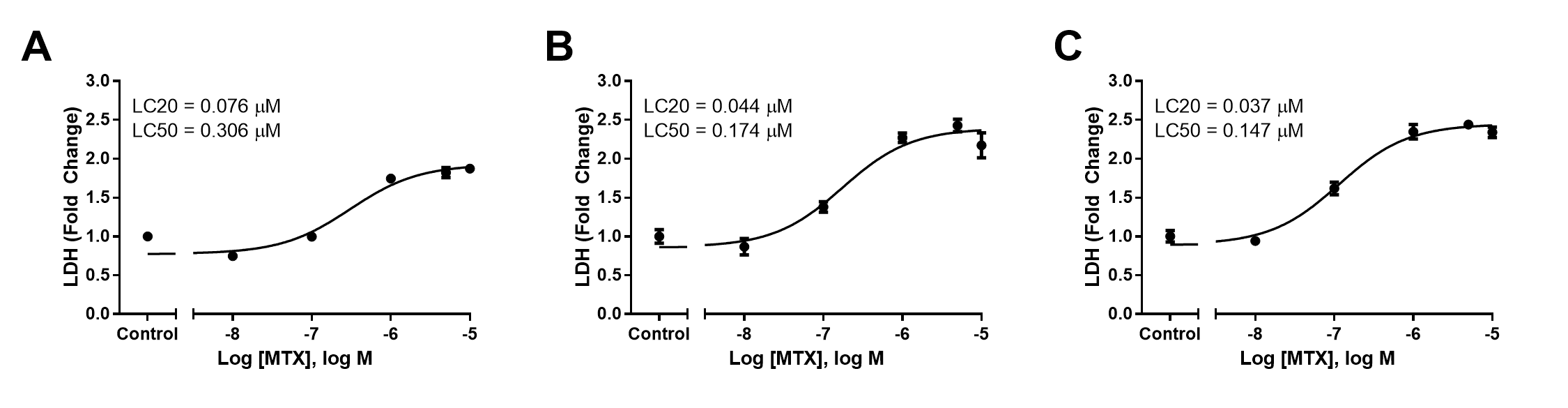

Supplement: S1 Fig — Dose response curves were fit to LDH fold change values for MTX treatments on days (A) 7, (B) 9, and (C) 11. Average values across the 3 days were used to obtain LC20 (0.052 μM) and LC50 (0.209 μM) concentrations used to conduct the present studies. Data represent the mean of the fold change + SEM (n = 3–5) for each concentration. (TIF) [file pone.0208958.s001.tif]

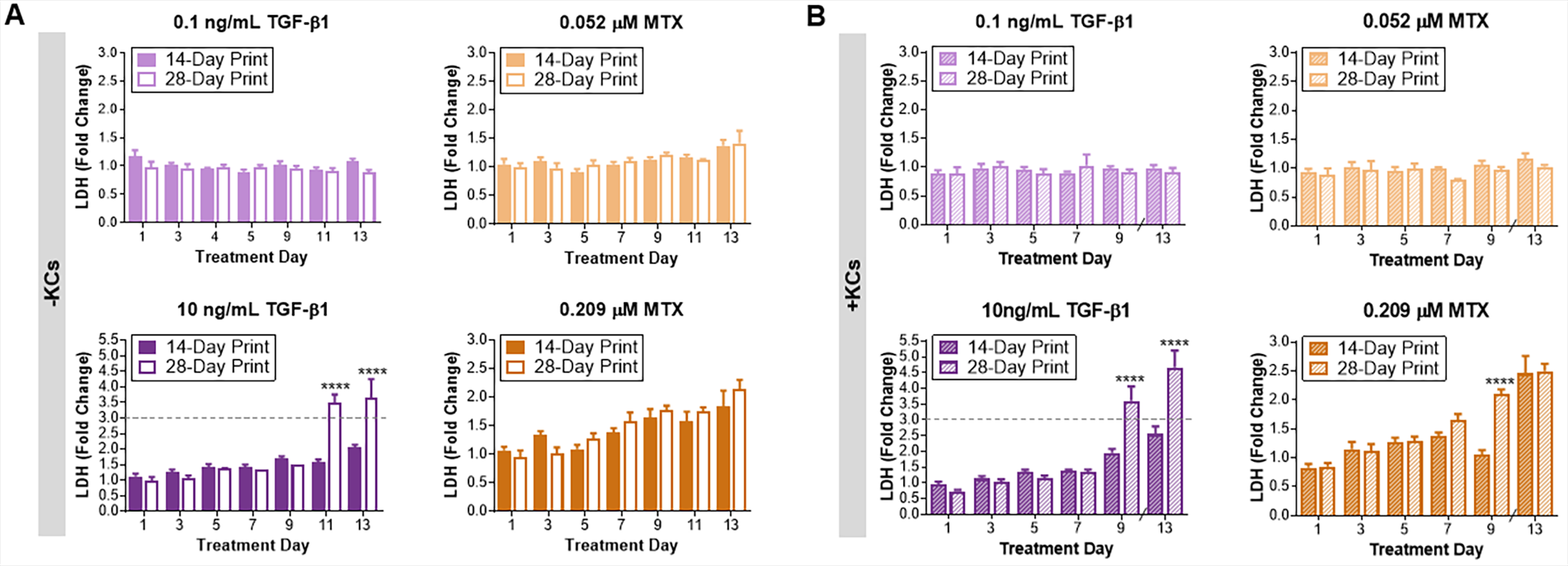

Supplement: S2 Fig — (A) LDH response profiles for all treatment groups for the standard (-KCs) tissue model. (B) LDH response profiles for all treatment groups for the modified (+KC) tissue model. Data represent the mean of the fold change + SEM (n = 5–7) for each model. Significance determined using two-way ANOVA with post hoc Dunnett’s multiple comparisons. *P < 0.05, **P < 0.01, ***P < 0.001, ****P < 0.0001. (TIF) [file pone.0208958.s002.tif]

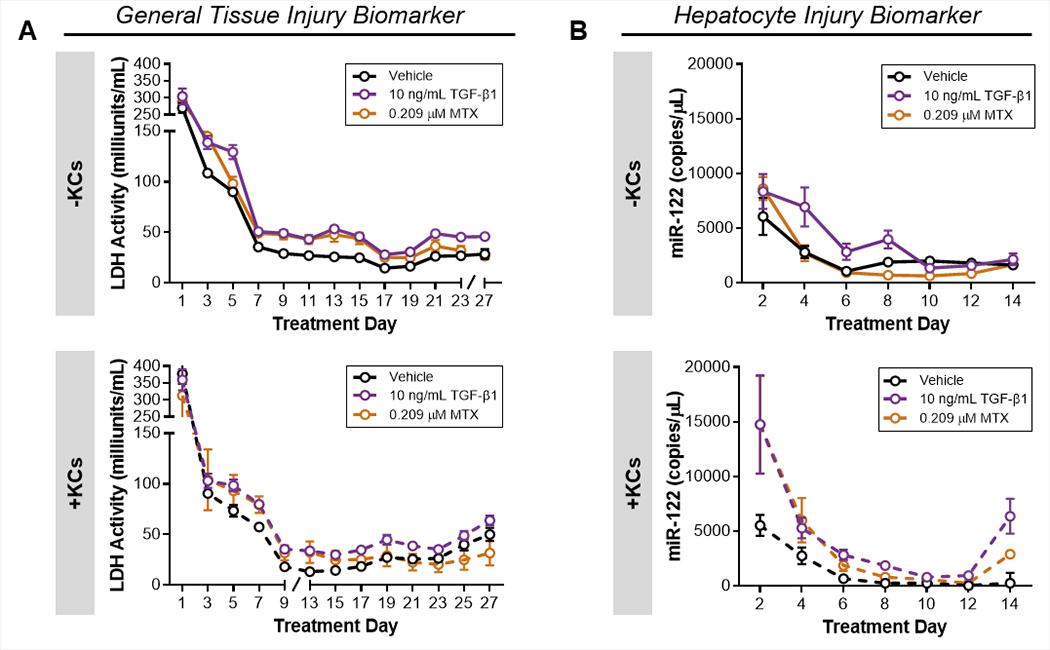

Supplement: S3 Fig — (A) LDH response profiles for 10 ng/mL TGF-β1 and 0.209 μM MTX for the standard (-KCs) and modified (+KCs) tissue model. (B) Corresponding miR-122 response profiles for each tissue model. Data represent the average raw vehicle values (black line) and average raw treatment values (colored lines) + SEM (n = 5–7). (TIF) [file pone.0208958.s003.tif]

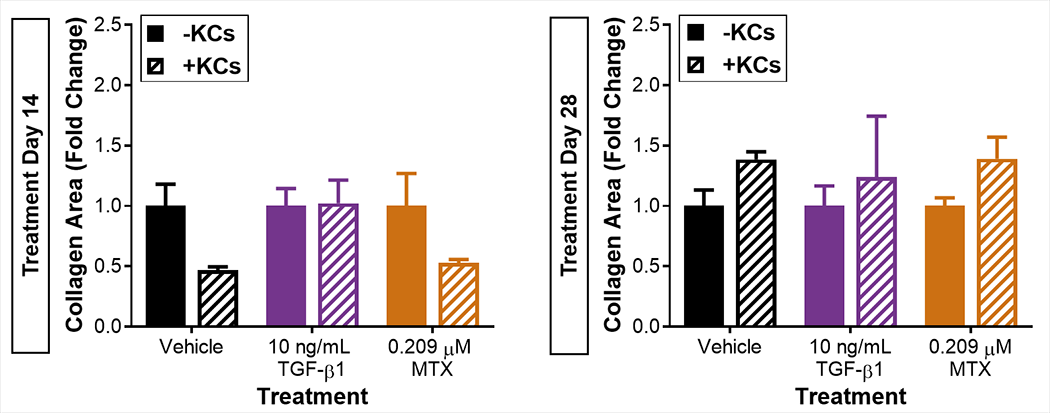

Supplement: S4 Fig — Data represent the mean of the fold change + SEM at each timepoint relative to effects observed in the standard tissue model (n = 2). At 14 days, KCs had a significant impact on collagen deposition (P< 0.01, two-way ANOVA). (TIF) [file pone.0208958.s004.tif]

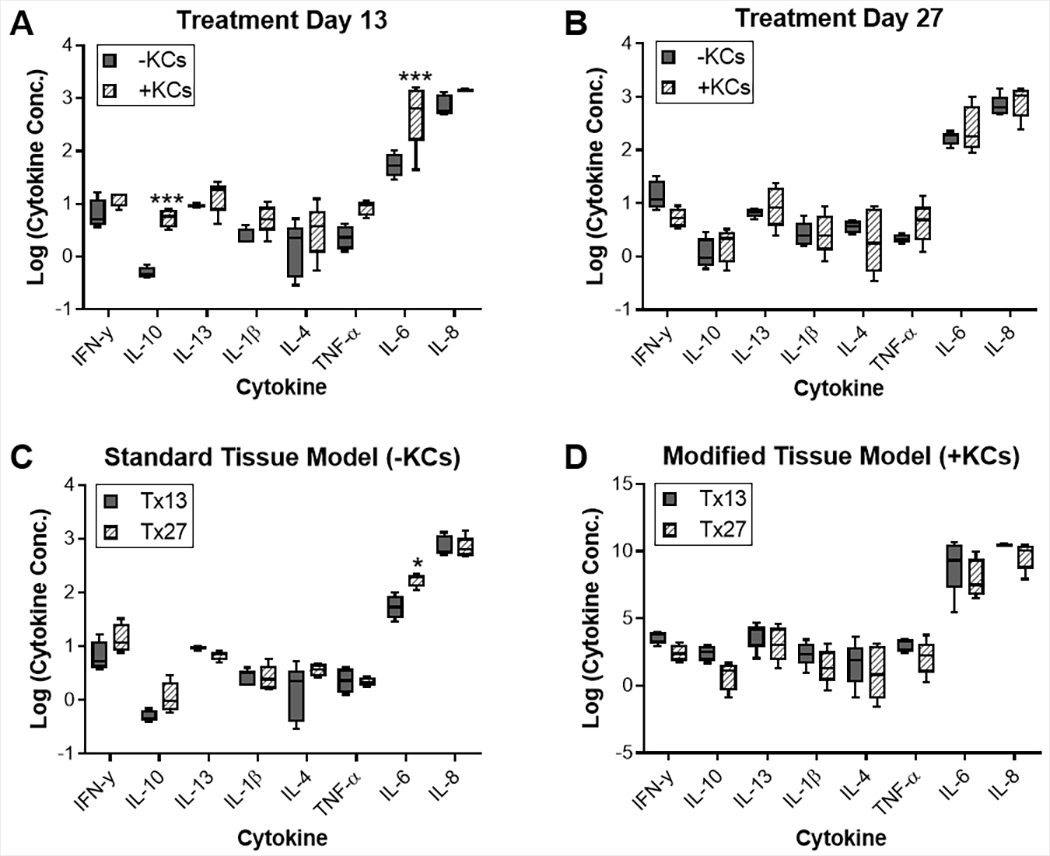

Supplement: S5 Fig — Box and whisker plots are used to describe the mean and range of the Log (Cytokine Concentration). Significance determined using two-way ANOVA with post hoc Sidak’s multiple comparisons. *P < 0.05 and ***P < 0.001. KCs had a significant impact on cytokine profiles at (A) treatment day 13 (P < 0.0001, two-way ANOVA) but not at (B) treatment day 27. Time had a significant impact on cytokine profiles in the (C) standard tissue model (P < 0.01, two-way ANOVA) and (D) modified tissue model (P < 0.001, two-way ANOVA). (TIF) [file pone.0208958.s005.tif]

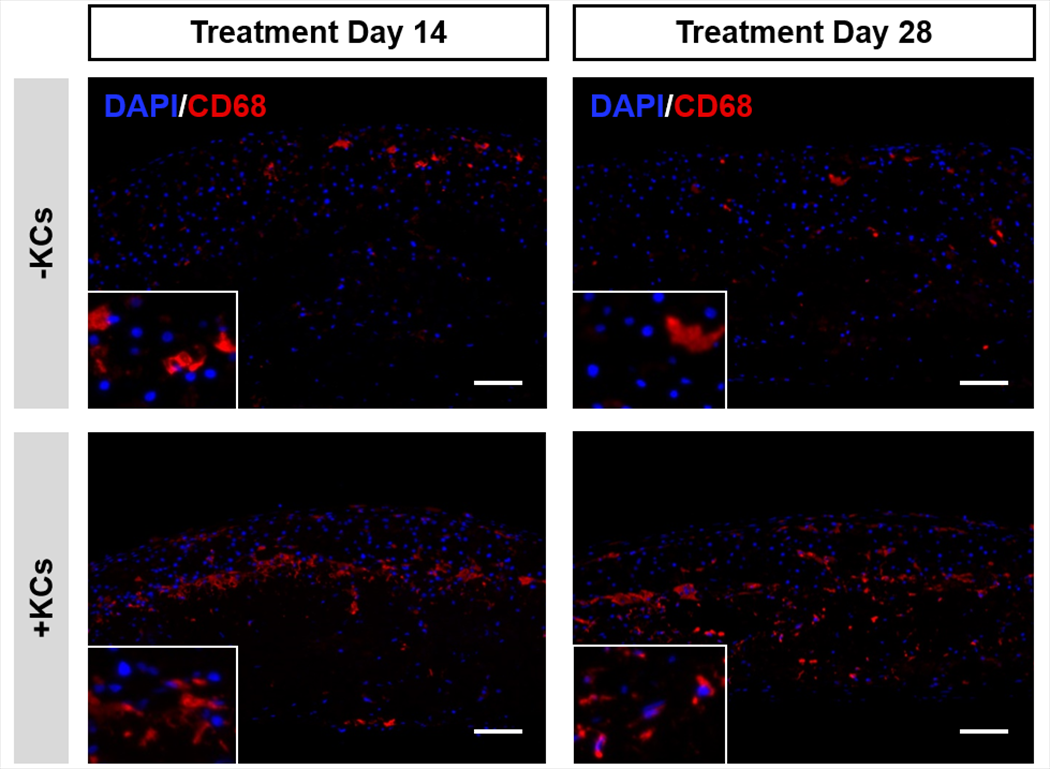

Supplement: S6 Fig — Representative photomicrographs of vehicle-treated tissue for the standard (-KCs) and modified (+KCs) tissue model. CD68 (red) was used to label KCs within the tissues. Scale bar = 100 μm. (TIF) [file pone.0208958.s006.tif]
